# Supplementary material for: Copper stress upregulates oxidative stress response, histidine production, and iron acquisition genes in Escherichia coli
Source: FEMS Microbes. 2026 Sep 17;7:xtag050. doi: 10.1093/femsmc/xtag050 (PMC13618251; doi:10.1093/femsmc/xtag050)
Supplement: Supplementary file 1 [file xtag050_supplemental_files.zip › FEMSMC-2026-030.R1 supplementary data - reviews and author response.docx]

**Reviews of manuscript FEMSMC-2026-030**

**Reviewer 1**

**Recommendation: Major Revision**

Ainelo et al. profile the transcriptional response of E. coli MG1655 to growth-slowing (2 mM) and near-lethal (8 mM) ionic copper by RNA-seq, reporting induction of canonical copper-homeostasis genes, oxidative stress defenses, histidine/methionine/arginine biosynthesis, and an extensive iron-acquisition program, alongside repression of biofilm/adhesion (2 mM) and anaerobic energy metabolism (8 mM) genes. They additionally evaluate, and ultimately reject, a commercial promoter–GFP library as a copper-screening tool. The dataset is broad, the manuscript is clearly written, and the candid reporting of negative results (iron supplementation, the promoter library, the quenching controls) is a genuine strength. The descriptive findings—which genes change under copper—are generally well supported. However, several issues in statistical reporting, experimental design, and the interpretive (mechanistic) conclusions should be addressed before the work is suitable for publication.

<Major comments>

1. RNA-seq replication and sample structure are not reported. The Methods describe pooling culture from microplate wells per condition but do not state the number of biological replicates sequenced, nor whether the pooling is technical or biological. A Padj threshold presupposes replication, yet n is given nowhere. Please state the replicate number and provide a sample-level quality overview (PCA or correlation heatmap) so that reproducibility and between-condition separation can be assessed.

2. The transcriptome is not validated by an independent method. The intended validation (promoter library) failed, and the biosensor confirms only CueR activation, not the broader response. With roughly one-fifth of the genome called differentially expressed at 2 mM, confirmation of a representative gene panel by qRT-PCR is warranted, particularly for the less-expected categories (gad repression, arginine biosynthesis, lipid A).

3. Several conclusions outrun the supporting data. The descriptive results are sound, but a number of mechanistic conclusions rest on transcript-level data alone:

– Iron. The conclusion that iron-acquisition induction “does not reflect true iron scarcity” overreaches. Figure 3 supports only that external iron is not growth-limiting. The induced Fur regulon (ent/fep/fec/feo) is the canonical signature of reduced Fur-accessible iron, which can fall while total iron is unchanged (consistent with the cited Casanova-Hampton). Please reframe around functional/regulatory iron limitation rather than dismissing it.

– Histidine and amino acids. That intracellular chelation is the “main benefit” of histidine induction is a transcript-only inference; excluding a buffering role does not establish chelation. No histidine measurement or biosynthesis-mutant copper MIC is provided. The medium also contains casamino acids, so His/Met/Arg are already supplied—induction of their biosynthesis despite supplementation should be addressed explicitly.

– Anaerobic metabolism. Repression of anaerobic/hydrogenase genes at 8 mM is interpreted via Fe–S disruption, but the cultures are aerobic, where these genes are already low; copper-specificity is unclear and should be noted.

– Functional claims. The gad/glutamate-conservation hypothesis is a multi-step speculation with no direct data (appropriately hedged), and “less inclined to settle on surfaces” is inferred without a biofilm assay.

4. Growth-rate confounding is not addressed. Copper slows (2 mM) or arrests (8 mM) growth relative to the faster-growing control. A fraction of the differentially expressed genes—especially repressed anabolic/translational categories—may reflect growth rate rather than copper-specific regulation. This should be acknowledged and, where possible, separated.

5. The advance over prior microarray studies is asserted but not quantified. No direct comparison of differentially expressed gene lists with Yamamoto & Ishihama (2005) or Kershaw et al. (2005) is provided. A concise overlap/novelty comparison would substantiate the “comprehensive, genome-wide” framing; the authors should also confirm whether this is the first RNA-seq characterization of copper stress in E. coli, or compare to existing RNA-seq datasets.

6. The promoter-library section is disproportionately weighted, and the quenching control lacks a positive case. Two of six figures concern a commercial product’s failure; the cautionary value is real, but the section competes with the RNA-seq as a co-primary result and could be streamlined. Critically, Figure 5 contains no positive control showing the assay can detect quenching when it occurs—every condition is negative—so it cannot distinguish a true absence of quenching from insufficient sensitivity. This conflicts with the cited Bourge et al. (2015), who report near-complete quenching at ~0.4 mM Cu, whereas no effect is seen here even at 4 mM. An in vitro positive control and a discussion of GFP-variant differences (GFPmut2 vs ST-GFP) and the likely low intracellular free-copper level would resolve this.

<Minor comments>

1. cusC vs cusS (p. 12). The 2293-fold / log2FC = 11.2 induction is attributed to cusS in the text but to cusC in Figure 4 (also the promoter discussed). Please reconcile; the value is also cited to Supplementary Figure 1 (the MIC assay) rather than to the DE gene list.

2. comR (ycfQ) is called “possibly random fluctuation” although it passes the authors’ own significance cutoff. Treat it as a real (if modest) signal, or justify exclusion on grounds other than chance.

3. The gad repression is framed as “unexpected” because copper acidifies the medium, but Figure 6 shows essentially no acidification in the MOPS medium used for RNA-seq (6.7 → 6.7 at 2 mM). The framing rests on LB, not on the RNA-seq condition.

4. Internal count inconsistencies. Oxidative stress genes total 12 (shared) + 5 (8 mM only) = 17 at 8 mM in Results (p. 10) but are stated as 12 at 8 mM in the Discussion (p. 16); lipid A is stated as 10 genes, but only 9 are listed.

5. 2–8 mM ionic copper is high relative to most antimicrobial-copper contexts; the translational framing (healthcare-associated infections) should be tempered to match what was tested. A single 30-min timepoint captures only the acute response and should be noted as a limitation.

6. A viability/CFU check at the 30-min sampling point would clarify whether the 8 mM transcriptome reflects a live response or a partial death signature.

7. Typos. Standardize gene names (bhsA is written as bshA; sodA as “soda”) and correct typos (“copper oxydative stress” in keywords; “signal to noise ration”; “fluoresence”; “errors bars”; “in in buffered MOPS”).

**Reviewer 2: Hoan Ngo**

**Recommendation: Minor Revision**

Ainelo et al has performed RNA sequencing to investigate changes in transcriptome of E. coli in response to inhibitory concentrations of copper. They also validated the data using a genome-wide GFP-based promoter library. In general, this study provides a general understanding on mechanisms regarding copper stress response in bacteria.

I have some minor suggestions on the manuscript.

1. Since the authors have checked iron deficiency and pH in bacteria exposed to copper, it would be good if the authors also test, in their own system, oxidative stress generated by copper by using the fluorescent dye DCFHA or the growth of bacteria treated with copper and oxidative stress scavenger thiourea.

2. Fig. 1B should go before Fig. 1A, based on the order it was mentioned.

3. Supplementary file 2 should go before Supplementary file 1, based on the order it was mentioned.

**Associate Editor Recommendation: Major Revision**

**EIC Decision: Major Revision**

**Author's Response to Decision Letter for (FEMSMC-2026-030)**

We thank the reviewers for the many insightful comments and important notes. We have considered the valuable input and present a revised version of the manuscript.

Please find our point-by-point responses to the comments below. The revisions in the main text are shown as tracked changes.

Reviewer: 1

Comments to the Author

Ainelo et al. profile the transcriptional response of E. coli MG1655 to growth-slowing (2 mM) and near-lethal (8 mM) ionic copper by RNA-seq, reporting induction of canonical copper-homeostasis genes, oxidative stress defenses, histidine/methionine/arginine biosynthesis, and an extensive iron-acquisition program, alongside repression of biofilm/adhesion (2 mM) and anaerobic energy metabolism (8 mM) genes. They additionally evaluate, and ultimately reject, a commercial promoter–GFP library as a copper-screening tool. The dataset is broad, the manuscript is clearly written, and the candid reporting of negative results (iron supplementation, the promoter library, the quenching controls) is a genuine strength. The descriptive findings—which genes change under copper—are generally well supported. However, several issues in statistical reporting, experimental design, and the interpretive (mechanistic) conclusions should be addressed before the work is suitable for publication.

1. RNA-seq replication and sample structure are not reported. The Methods describe pooling culture from microplate wells per condition but do not state the number of biological replicates sequenced, nor whether the pooling is technical or biological. A Padj threshold presupposes replication, yet n is given nowhere. Please state the replicate number and provide a sample-level quality overview (PCA or correlation heatmap) so that reproducibility and between-condition separation can be assessed.

We have added to the Materials and Methods that four samples from three fully independent experiments were used in this analysis. Two samples are not entirely independent, as they were collected on the same day, although derived from different stocks and grown in independently prepared media. The growth of their technical replicates is presented in Figure 1B.

We have added the heatmap as Supplementary Figure 2.

2. The transcriptome is not validated by an independent method. The intended validation (promoter library) failed, and the biosensor confirms only CueR activation, not the broader response. With roughly one-fifth of the genome called differentially expressed at 2 mM, confirmation of a representative gene panel by qRT-PCR is warranted, particularly for the less-expected categories (gad repression, arginine biosynthesis, lipid A).

Indeed, the whole transcriptome is not separately validated.

Firstly, we would like to emphasize that the promoter library was not intended as a validation but was explored as an alternative bacterial cell-based method. The expected benefit of the library would have been the flexibility to perform targeted expression screens (using a subset of promoters) in different growth conditions without the need for a full transcriptome.

Secondly, we explored the actual need for independent experimental validation and found that generally, RNAseq based transcriptomics can be considered reliable on their own when the limits of what is considered significant are well adjusted (Coenye, 2021). Additionally, we draw our main conclusions from the results of enrichment analyses, which look at larger trends in the dataset and reduce the risk of over-interpreting single gene results.

Following the reviewer’s suggestions, we have toned down the discussion sections that consider the less-supported categories as they can indeed be read as too speculative.

3. Several conclusions outrun the supporting data. The descriptive results are sound, but a number of mechanistic conclusions rest on transcript-level data alone:

– Iron. The conclusion that iron-acquisition induction “does not reflect true iron scarcity” overreaches. Figure 3 supports only that external iron is not growth-limiting. The induced Fur regulon (ent/fep/fec/feo) is the canonical signature of reduced Fur-accessible iron, which can fall while total iron is unchanged (consistent with the cited Casanova-Hampton). Please reframe around functional/regulatory iron limitation rather than dismissing it.

We have revised the relevant paragraph.

– Histidine and amino acids. That intracellular chelation is the “main benefit” of histidine induction is a transcript-only inference; excluding a buffering role does not establish chelation. No histidine measurement or biosynthesis-mutant copper MIC is provided. The medium also contains casamino acids, so His/Met/Arg are already supplied—induction of their biosynthesis despite supplementation should be addressed explicitly.

As suggested, we measured the copper MIC for several histidine biosynthesis mutants and found no difference compared to the wild-type. The relevant discussion paragraph has been updated to reduce speculativity.

– Anaerobic metabolism. Repression of anaerobic/hydrogenase genes at 8 mM is interpreted via Fe–S disruption, but the cultures are aerobic, where these genes are already low; copper-specificity is unclear and should be noted.

We have noted this in the discussion.

– Functional claims. The gad/glutamate-conservation hypothesis is a multi-step speculation with no direct data (appropriately hedged), and “less inclined to settle on surfaces” is inferred without a biofilm assay.

Indeed, these are hypotheses put forth as discussion, not concrete results.

We have adjusted the text to make it clearer that these are possibilities and not facts.

4. Growth-rate confounding is not addressed. Copper slows (2 mM) or arrests (8 mM) growth relative to the faster-growing control. A fraction of the differentially expressed genes—especially repressed anabolic/translational categories—may reflect growth rate rather than copper-specific regulation. This should be acknowledged and, where possible, separated.

We have acknowledged this possibility at the discussion of repressed genes.

5. The advance over prior microarray studies is asserted but not quantified. No direct comparison of differentially expressed gene lists with Yamamoto & Ishihama (2005) or Kershaw et al. (2005) is provided. A concise overlap/novelty comparison would substantiate the “comprehensive, genome-wide” framing; the authors should also confirm whether this is the first RNA-seq characterization of copper stress in E. coli, or compare to existing RNA-seq datasets.

Indeed, this is not the first time ever when RNA-seq has been performed on E. coli with added copper, however to our knowledge it is the first that studies stressful levels of copper. The previous works we could find concern much lower copper concentrations or study other variables such as evolution:

(Zhang et al., 2019) used 5 μM copper, (Darwiche et al., 2025) used 15 μM copper, and

(Salazar-Alemán et al., 2025) published a preprint where they used 39 μM copper.

Those micromolar concentrations do not represent a meaningful copper stress to E. coli and therefore are not a good comparison to our work. The microarray studies used 0.5 mM (Yamamoto & Ishihama) and 2 mM (Kershaw et al.) copper, which are better references.

There is a work (Boyd et al., 2022) that used a more comparable copper concentration of 0.47 mM in a RNA-seq experiment but only studied the effects of copper after 37 days of adaptive evolution. While interesting, this does not necessarily reflect the immediate response to a copper shock that we were interested in.

Comparisons to the microarray studies have been added to the text. In the case of the paper by Kershaw et al., they do report a total of 340 genes as being differentially regulated upon copper addition, however a comprehensive list is not given, which precludes drawing a comparison with our results.

6. The promoter-library section is disproportionately weighted, and the quenching control lacks a positive case. Two of six figures concern a commercial product’s failure; the cautionary value is real, but the section competes with the RNA-seq as a co-primary result and could be streamlined. Critically, Figure 5 contains no positive control showing the assay can detect quenching when it occurs—every condition is negative—so it cannot distinguish a true absence of quenching from insufficient sensitivity. This conflicts with the cited Bourge et al. (2015), who report near-complete quenching at ~0.4 mM Cu, whereas no effect is seen here even at 4 mM. An in vitro positive control and a discussion of GFP-variant differences (GFPmut2 vs ST-GFP) and the likely low intracellular free-copper level would resolve this.

The data in Figure 5 is not inherently in conflict with Bourge et al (2015). In their use, the plant cells are permeable to copper ions, as is required for the click labelling chemistry they use. In our case, E. coli cells can utilize the copper detoxification pathways to prevent the actual intracellular copper concentration from rising to comparable millimolar levels.

The reasoning behind this particular experiment was not to fully demonstrate copper fluorescence quenching in E. coli cells, rather to determine whether it was the copper itself that was holding back the GFP assay. According to the collected data, we can only say that no, copper or the other antimicrobials are not particularly at fault for the low sensitivity.

In our view, demonstrating Cu quenching GFP fluorescence in vitro would yield results that do little to address the question of practical copper/GFP interactions in E. coli cells, precisely due to the likely low intracellular free Cu levels.

1. cusC vs cusS (p. 12). The 2293-fold / log2FC = 11.2 induction is attributed to cusS in the text but to cusC in Figure 4 (also the promoter discussed). Please reconcile; the value is also cited to Supplementary Figure 1 (the MIC assay) rather than to the DE gene list.

We have corrected the text.

2. comR (ycfQ) is called “possibly random fluctuation” although it passes the authors’ own significance cutoff. Treat it as a real (if modest) signal, or justify exclusion on grounds other than chance.

We found that ComR repression is generally released upon copper addition, which resolves the apparent discrepancy in the first version of the paper. This has been added to the text.

3. The gad repression is framed as “unexpected” because copper acidifies the medium, but Figure 6 shows essentially no acidification in the MOPS medium used for RNA-seq (6.7 → 6.7 at 2 mM). The framing rests on LB, not on the RNA-seq condition.

We have trimmed the results paragraph and amended the corresponding discussion paragraph.

4. Internal count inconsistencies. Oxidative stress genes total 12 (shared) + 5 (8 mM only) = 17 at 8 mM in Results (p. 10) but are stated as 12 at 8 mM in the Discussion (p. 16); lipid A is stated as 10 genes, but only 9 are listed.

We have corrected the typo in the discussion.

5. 2–8 mM ionic copper is high relative to most antimicrobial-copper contexts; the translational framing (healthcare-associated infections) should be tempered to match what was tested. A single 30-min timepoint captures only the acute response and should be noted as a limitation.

The healthcare-associated infections are mentioned in the introduction as a general reasoning for studying the effects of copper on bacteria. In our view, it is not presented as the overarching framework for our research.

It is true that the concentrations used are high, however they were selected based on their effect on bacterial growth in the tested conditions. We agree that a 30-minute timepoint is a snapshot and the text has been edited to reflect that.

6. A viability/CFU check at the 30-min sampling point would clarify whether the 8 mM transcriptome reflects a live response or a partial death signature.

We have uploaded a close-up of figure 1A showing the growth of technical replicates of the exact sample used for RNA-seq (same inoculum, measured in a separate well). At 30 minutes, the number of cells is still increasing. Even at 8 mM, cell numbers continue to increase at 5 hours (Figure 1A). Therefore, we assume that the observed changes predominantly reflect a response of actively growing cells

7. Typos. Standardize gene names (bhsA is written as bshA; sodA as “soda”) and correct typos (“copper oxydative stress” in keywords; “signal to noise ration”; “fluoresence”; “errors bars”; “in in buffered MOPS”).

The text has been corrected.

Reviewer: 2

Comments to the Author

Ainelo et al has performed RNA sequencing to investigate changes in transcriptome of E. coli in response to inhibitory concentrations of copper. They also validated the data using a genome-wide GFP-based promoter library. In general, this study provides a general understanding on mechanisms regarding copper stress response in bacteria.

I have some minor suggestions on the manuscript.

1. Since the authors have checked iron deficiency and pH in bacteria exposed to copper, it would be good if the authors also test, in their own system, oxidative stress generated by copper by using the fluorescent dye DCFHA or the growth of bacteria treated with copper and oxidative stress scavenger thiourea.

We have also considered this type of experiment. However, one important limitation is that copper itself is a redox-active metal (Tsang et al., 2021).

In addition, commonly used ROS scavengers are not suitable in this context, as many of them can interact with and bind copper, thereby potentially confounding the interpretation of the results. For example, thiourea (Dömling et al., 2025), mannitol (Doleźal et al., 1973), DMSO (Šimunková and Malček, 2020) and glutathione (Aliaga et al., 2016) have all been reported to bind copper. Therefore we did not pursue these measurements.

2. Fig. 1B should go before Fig. 1A, based on the order it was mentioned.

Rearranged as suggested.

3. Supplementary file 2 should go before Supplementary file 1, based on the order it was mentioned.

Rearranged as suggested.

References:

Aliaga, M.E., López-Alarcón, C., Bridi, R., and Speisky, H. (2016) Redox-implications associated with the formation of complexes between copper ions and reduced or oxidized glutathione. Journal of inorganic biochemistry 154: 78–88.

Boyd, S.M., Rhinehardt, K.L., Ewunkem, A.J., Harrison, S.H., Thomas, M.D., and Graves, J.L. (2022) Experimental Evolution of Copper Resistance in Escherichia coli Produces Evolutionary Trade-Offs in the Antibiotics Chloramphenicol, Bacitracin, and Sulfonamide. Antibiotics 11: 711.

Coenye, T. (2021) Do results obtained with RNA-sequencing require independent verification? Biofilm 3: 100043.

Darwiche, L., Rodriguez-Bornot Carlos A., Ingrassia Rebecca A., Loccisano Max J., Waldschmidt Gray, and Goff Jennifer L. (2025) The molecular basis of the synergistic toxicity of nickel and copper, common environmental co-contaminants. Applied and Environmental Microbiology 91: e01627-25.

Doleźal, J., Klausen, K., and Langymyhr, F. (1973) Studies in the complex formation of metal ions with sugars: Part I. The complex formation of cobalt (II), cobalt (III), copper (II) and nickel (II) with mannitol. Analytica Chimica Acta 63: 71–77.

Dömling, A., Klein, D., Manoharan, S., and Mohr, F. (2025) Structural variety in coinage metal complexes containing thiourea-derivatives. Chemistry Letters 54: upaf116.

Salazar-Alemán, D.A., McGibbon, A., and Turner, R.J. (2025) Contrasts and similarities in the transcriptomic response to antimicrobial coinage metals in *Escherichia coli*. bioRxiv 2025.08.12.669831.

Šimunková, M., and Malček, M. (2020) Dimethyl sulfoxide as a strongly coordinating solvent: 3′, 4′-dihydroxyflavone-Cu (II)-DMSO system case study. Acta Chimica Slovaca 13: 38–48.

Tsang, T., Davis, C.I., and Brady, D.C. (2021) Copper biology. Current Biology 31: R421–R427.

Zhang, S., Wang, Y., Song, H., Lu, J., Yuan, Z., and Guo, J. (2019) Copper nanoparticles and copper ions promote horizontal transfer of plasmid-mediated multi-antibiotic resistance genes across bacterial genera. Environment International 129: 478–487.

**Reviews of manuscript FEMSMC-2026-030.R1**

**Reviewer 1**

**Recommendation: Accept**

The authors have addressed the previous concerns thoroughly. I recommend acceptance.

**Reviewer 2: Hoan Ngo**

**Recommendation: Accept**

The authors have addressed all of my concerns. And the revisions strengthens the mannuscript.

**Associate Editor Recommendation: Accept**

**EIC Decision: Accept**
