# Supplementary material for: Copper stress upregulates oxidative stress response, histidine production, and iron acquisition genes in Escherichia coli
Source: FEMS Microbes. 2026 Sep 17;7:xtag050. doi: 10.1093/femsmc/xtag050 (PMC13618251; doi:10.1093/femsmc/xtag050)
Supplement: Supplementary file 1 [file xtag050_supplemental_files.zip › Supplementary data.docx]

## **Supplementary data**

**Supplementary Figure 1.** Determining *E. coli* MG1655 MIC for the growth of transcriptome samples. The error bars represent mean ± SD of one experiment with four replicates.

**Supplementary Figure 2.** Heatmap showing differential gene expression patterns across samples. Red indicates upregulated genes, white represents no change, and blue indicates downregulated genes. The color scale is based on z‑scores, allowing direct comparison of expression levels across genes and samples.

**Supplementary Figure 3.** Determination of the MIC of *E. coli* MG1655 carrying the pUA66‑fes plasmid (Km⁺) in MOPS (**A**) and LB (**B**). Error bars represent mean ± 95% CI of four biological experiments.
